# A Cross-Sectional Study on Elevated Serum Calcitonin Gene-Related Peptide Levels in Rheumatoid Arthritis Patients and Their Relationship with Disease Activity

**DOI:** 10.5152/ArchRheumatol.2026.10704

**Published:** 2026-01-16

**Authors:** Merve Dede Akpınar, Safinaz Ataoğlu, Şengül Cangür

**Affiliations:** 1 Department of Physical Medicine and Rehabilitation, Bitlis State Hospital, Bitlis, Türkiye; 2 Department of Physical Medicine and Rehabilitation, Düzce University Medical Faculty, Düzce, Türkiye; 3 Department of Biostatistics and Medical Informatics, Düzce University Medical Faculty, Düzce, Türkiye

**Keywords:** Calcitonin gene-related peptide, DAS-28–ESR, disease activity, rheumatoid arthritis

## Abstract

**Background/Aims:**

This study aims to assess serum levels of calcitonin gene-related peptide (CGRP) in patients with rheumatoid arthritis (RA), compare these levels with those of healthy controls, and analyze their correlation with disease activity.

**Materials and Methods:**

This cross-sectional, case-controlled study involved 80 individuals diagnosed with RA based on the American College of Rheumatology/The European Alliance of Associations for Rheumatology (ACR/EULAR) 2010 criteria and 40 healthy individuals serving as the control group. Demographic data, along with clinical and laboratory findings of the participants, were documented for analysis. C-reactive protein (CRP), CGRP, and erythrocyte sedimentation rate (ESR) levels were measured in both the RA and control groups. Disease activity among patients with RA was assessed using the Visual Analog Scale (VAS), Disease Activity Score-28 (DAS-28), and Health Assessment Questionnaire (HAQ). Logistic regression was performed to identify significant predictors, and the optimal cut-off value was established through receiver operating characteristic (ROC) analysis.

**Results:**

The median serum CGRP level in patients with RA (91.1 pg/mL) was significantly higher than that of the control group (40.8 pg/mL) (*P* < .001). The median serum CGRP level was significantly greater in seropositive patients with RA (118.7 pg/mL) compared to seronegative patients (66.1 pg/mL) (*P* = .017). The ROC analysis identified 61.78 pg/mL as the optimal CGRP cut-off to distinguish patients with RA from controls, with 70% sensitivity and 87.5% specificity (Area Under the Curve (AUC): 0.839, *P* < .001). A significant correlation was observed between CGRP levels in patients with RA and the scores of DAS-28, HAQ, VAS-Patient, and VAS-Physician (*P* = .001, *P* = .006, *P* < .001, *P* = .001). Logistic regression analyses demonstrated that increases in CGRP (*P* = .002) and CRP (*P* = .011), as well as CGRP (*P* = .001) and ESR (*P* < .001), were each significantly associated with higher odds of active disease.

**Conclusion:**

The findings suggest that higher serum CGRP levels may contribute to the pathophysiology of RA, potentially indicating disease progression, joint damage, and supporting prognosis in the management of RA.

**Main Points**

- Patients with rheumatoid arthritis have significantly higher serum CGRP levels compared to healthy controls, with especially higher levels observed in seropositive patients with RA.
- Serum CGRP levels show a significant positive correlation with RA disease activity and severity measures, including DAS-28, HAQ, VAS-Patient, and VAS-Physician scores.
- CGRP demonstrates good diagnostic performance in distinguishing patients with RA from healthy individuals and is independently associated with active disease, suggesting its potential role for disease activity and prognosis in RA.

## Introduction

Rheumatoid arthritis is a chronic autoimmune disease marked by synovial inflammation, joint damage, cartilage loss, and bone erosion. It affects about 1% of the global population, with an incidence of approximately 0.36% in Türkiye.^1^ Rheumatoid arthritis predominantly affects females, with a frequency 3-4 times higher than in males.^2^ The disease is linked to socio-economic challenges, systemic complications, progressive disability, and increased mortality. People with RA often experience physical, psychological, and social impacts, leading to a diminished quality of life. The economic burden of RA in the US reaches $19.3 billion each year, accompanied by extra costs related to lower quality of life and early mortality.^3^

The underlying factors of RA are not completely clear, but it is considered to develop from a mix of genetic risk and external factors.^2^ While some cases may show mild courses with spontaneous remission, others may escalate rapidly, leading to severe disability. Key factors influencing the disease’s progression include the presence of rheumatoid nodules, joint destruction, positive rheumatoid factor (RF), elevated erythrocyte sedimentation rate (ESR), positive anti-cyclic citrullinated peptide (CCP) test, depressive symptoms, and specific genetic markers.^4,5^

Evaluating disease activity is essential for early treatment with disease-modifying antirheumatic drugs (DMARDs) to prevent RA’s progression.^6^ Disease activity is measured using various scoring methods, including the Disease Activity Score-28 (DAS-28), the Clinical Disease Activity Index, the Simplified Disease Activity Index, and the Health Assessment Questionnaire Disability Index.^7,8^ The DAS-28 remains the most commonly used metric, although it has limitations, such as the subjectivity of joint counts and the need for acute phase reactants.^9^ Moreover, the DAS-28 index includes patient-reported parameters, which means it can be influenced by factors such as pain perception, psychological status, and sleep quality. These factors may introduce variability in the assessment, as they are subjective and can be affected by the individual’s emotional and physical well-being. Therefore, there is a growing interest in identifying biomarkers for easier and more efficient disease assessment.^10^

Calcitonin gene-related peptide (CGRP) is a neuropeptide that is involved in pain perception, inflammatory responses, and neurogenic signaling and has been identified as a possible biomarker for arthritis.^11,12^ Calcitonin gene-related peptide is found in sensory nerves within joint synovium, periosteum, and subchondral bone, and its accumulation in synovial fluid has been linked to pain and disease activity in RA.^13^ Increased CGRP levels in synovial fluid are thought to play a role in arthritis pathology, positioning CGRP as a potential focus for treatments designed to regulate disease activity and enhance patient outcomes.^14^

Accordingly, this study was designed to compare serum CGRP levels between patients with RA and healthy controls, and to investigate the association between CGRP concentrations and clinical measures of disease activity.

## Materials and Methods

This cross-sectional, case-controlled study was conducted between November 2021 and May 2022 at the Düzce University medical faculty, specifically within the Department of Physical Medicine and Rehabilitation. Prior to initiating the study, ethical approval was granted by the Düzce University Faculty of Medicine Non-Interventional Health Research Board, with the date of October 18, 2021, and the associated decision number being 2021/209, underscoring the adherence to ethical standards in medical research. The study protocol was prepared in accordance with the ethical principles outlined in the Declaration of Helsinki, which serves as a foundational guideline for research involving human participants. All participants were informed in detail about the study’s purpose and procedures, and written informed consent was obtained to ensure ethical compliance and respect for individual autonomy. Participants met the established ACR/EULAR 2010 RA classification criteria and were between 18 and 65 years of age, ensuring a clearly defined study population. In this study, a total of 120 subjects were selected using simple random sampling in accordance with the study protocol to obtain a clinically and statistically significant difference for CGRP (pg/mL), with a significance level of 5%, power of 80%, a medium effect size (Cohen’s d) of 0.55, and a ratio of the number of subjects in the groups of 2 : 1. The Cohen’s effect size used in this study was calculated based on the results of 2 similar clinical studies in the literature.^15,16^ For sample size calculation, G*Power 3.1.9.2 program was used. Age groups “18-35, 36-50, 51-65” and a 2 : 1 subject selection ratio were taken into account when selecting the patient and control groups. Accordingly, a patient was selected using a random number generator, and a random subject was selected for the control group based on the selected patient’s age group. Additionally, the seronegative or seropositive rate of patients was also taken into account in the selection of the patient group. The patient group comprised 80 patients with confirmed RA and 40 age-matched healthy volunteers serving as controls. While the groups were matched for age, they also showed comparable characteristics in terms of sex, marital status, occupation, and smoking habits, except for educational status, which differed between the groups. Forty of the patients with RA were seronegative, while 40 of them were seropositive. Exclusion criteria were pregnancy or lactation, acute or chronic infection, malignancy, secondary rheumatological disease accompanying RA, advanced renal failure, advanced liver failure, advanced chronic obstructive pulmonary disease, migraine, use of medications that affect CGRP levels, and lack of cooperation that prevents the patient from understanding the questionnaires.

Demographic information of the individuals (age, gender, education level, marital status, profession, and body mass index (BMI), ESR, C-reactive protein (CRP) levels, antinuclear antibody (ANA), anti-CCP, RF results, and medications used for RA were recorded. Six patients were not using medication, 28 patients were using conventional synthetic DMARD (csDMARD), 19 patients were using biological DMARD (bDMARD), and 27 patients were using csDMARD+bDMARD. Conventional synthetic DMARD treatment included hydroxychloroquine sulfate, salazopyrin, methotrexate, and leflunomide; bDMARD treatment included adalimumab, golimumab, etanercept, certolizumab, abatacept, tofacitinib, and secukinumab.

Individuals diagnosed with RA were systematically evaluated for several key clinical indicators, such as the duration of the disease, time to resolution of stiffness after waking, counts of tender and swollen joints, and the therapies administered during the course of the illness. In addition to these evaluations, all participants completed the HAQ, while VAS and DAS-28^9^ scores—specifically for patients with RA—were calculated and analyzed separately to ensure accuracy and reliability. This comprehensive evaluation strategy offers a thorough perspective on patient status and clinical experience, while also enhancing insight into the effectiveness of different therapeutic interventions and their effects on overall well-being in individuals affected by RA.

Based on DAS-28 scores, patients were initially categorized into 4 groups: remission (≤2.6), low activity (2.6-3.2), moderate activity (3.2-5.1), and high activity (≥5.1). For analytical clarity and to better examine the relationship between disease activity levels and various sociodemographic, clinical, and laboratory variables, individuals with RA were further classified into 2 subgroups: remission (n = 31) and active disease (n = 49), using the DAS-28–ESR composite index.

Patients with RF values <20 U/mL were classified as RF negative, and those with >20 U/mL as RF positive, based on standard kit values. Anti-CCP antibodies were used to classify patients as positive or negative.^17,18^

### Visual Analog Scale

The Visual Analog Scale (VAS) is employed to assess pain intensity and is measured by using a 10-centimeter horizontal line placed on plain paper, marked at 1 cm intervals from 0 to 10. Patients were instructed that the left end represented “no pain,” while the right end indicated “the most severe pain imaginable.” They were then asked to indicate their current pain level on this scale. The resulting VAS scores were recorded separately as VAS-Patient (self-assessed) and VAS-Physician (clinician-assessed).

### Health Assessment Questionnaire

The Health Assessment Questionnaire (HAQ) is a scale consisting of 8 subcategories and 20 questions evaluating daily living activities, limitations in the individual’s physical functions, and structural damage caused by the disease process. In such scoring, the use of assistive devices and assistance requested from another person are also considered. Participants were asked to give graded answers to the questions according to their ability to do the activity by using 0: “I can do it easily,” 1: “with some difficulty,” 2: “with great difficulty,” 3: “I cannot do it at all.” Each section was scored separately, and a single HAQ score, which varies from 0 to 3, was determined by averaging the scores of 8 sections.

### Determination of Serum Calcitonin Gene-Related Peptide Level

After fasting for 24 hours, 5 milliliters of blood were drawn from every participant, and these samples were then transferred into specialized biochemistry tubes that were specifically designed to contain separator gel, which serves a crucial purpose in the processing and analysis of the blood. Samples were kept at room temperature for 2 hours, centrifuged at 179 × g for 20 minutes using a NÜVE NF1200 centrifuge, and then stored frozen at −80°C until analysis. Serum CGRP levels were determined by enzyme-linked immunosorbent assay (ELISA) (ELK Biotech; ELK1037 Human CGRP ELISA) according to the manufacturer’s protocol. After adding the standard solution and sample according to the test procedure, it was incubated at 37°C for 80 minutes in a CO_2_ incubator (NÜVE EC 160). After incubation, the samples were analyzed, and absorbance was measured at 450 nm using an Epoch Microplate Spectrophotometer.

### Statistical Analysis

The statistical analysis for this research was conducted utilizing the statistical software program known as SPSS version 22 (IBM SPSS Corp.; Armonk, NY, USA), which is widely recognized for its robust analytical capabilities. The study will randomly select 120 individuals, with 80 in the experimental group and 40 in the control group. In order to evaluate the normality of continuous quantitative variables, the Shapiro–Wilk test was employed, while the Levene test was specifically utilized to assess the homogeneity of variances across different groups under investigation. To facilitate meaningful comparisons of quantitative variables between various groups, a series of statistical tests was implemented, including the Kruskal–Wallis test, the Mann–Whitney *U*-test, and the independent samples *t*-test, each serving distinct purposes depending on the data characteristics. Spearman’s correlation coefficient was used to assess the relationships between continuous variables, indicating the strength and direction of associations. Additionally, logistic regression analysis was performed to identify significant factors influencing the study outcomes. To establish the most appropriate cut-off value for CGRP levels, a thorough receiver operating characteristic (ROC) analysis was conducted, which enabled an evaluation of the diagnostic performance of this biomarker. Additionally, to investigate the relationships between categorical variables, a suite of statistical tests was employed, including the Fisher–Freeman–Halton test, which is a Pearson’s chi-square test and Fisher’s exact test, each chosen for its particular relevance to the data structure. In all statistical analyses, a *P*-value of less than .05 was considered statistically significant.

## Results

Table 1 summarizes the demographic characteristics of the study sample. Patients with RA showed significantly higher levels of CGRP, BMI, and CRP (*P* < .001 for all), as well as ESR (*P* = .002), compared to the control group. Calcitonin gene-related peptide concentrations were notably increased in patients who were seropositive (anti-CCP positive) and RF-positive (*P* = .017 and *P* = .012, respectively). However, no statistically relevant variation in CGRP levels was observed in relation to anti-CCP status, ANA results, smoking habits, or treatment approach (*P* > .05), as presented in Table 2.

Table 3 outlines the sociodemographic distribution across DAS-28–based disease activity. Individuals with high disease activity showed elevated HAQ, VAS-Patient, and VAS-Physician scores relative to those in the moderate group (*P* < .001 for all). In contrast, age, CGRP, CRP, ESR, and RF/anti-CCP positivity did not differ meaningfully between the 2 groups (*P* > .05).

Table 4 compares the remission and active RA groups according to DAS-28–ESR. Higher levels of CGRP, ESR, CRP, HAQ, VAS-Patient, and VAS-Physician (*P* < .001 for all), as well as RF positivity, were found to be associated with active RA. The result of the logistic regression model with CGRP and CRP indicated that a 1-unit increase in CGRP (*P* = .002) and CRP (*P* = .011) increased the likelihood of active disease by 1.013 and 2.651 times, respectively. Also, the result of the logistic regression model with CGRP and ESR showed that a 1-unit increase in CGRP (*P* = .001) ESR (*P* < .001) increased the likelihood of active disease by 1.014 and 1.097 times, respectively (Table 5). No significant logistic regression models were obtained for HAQ, RF, anti-CCP, and ANA (*P* > .05).

Correlation analysis demonstrated moderate positive associations between CGRP and DAS-28 (*P* = .001), HAQ (*P* = .006), VAS-Patient (*P* < .001), and VAS-Physician (*P* = .001), as detailed in Table 6. No significant correlations were identified between CGRP and CRP, ESR, RA disease duration, or morning stiffness.

**Figure 1.**
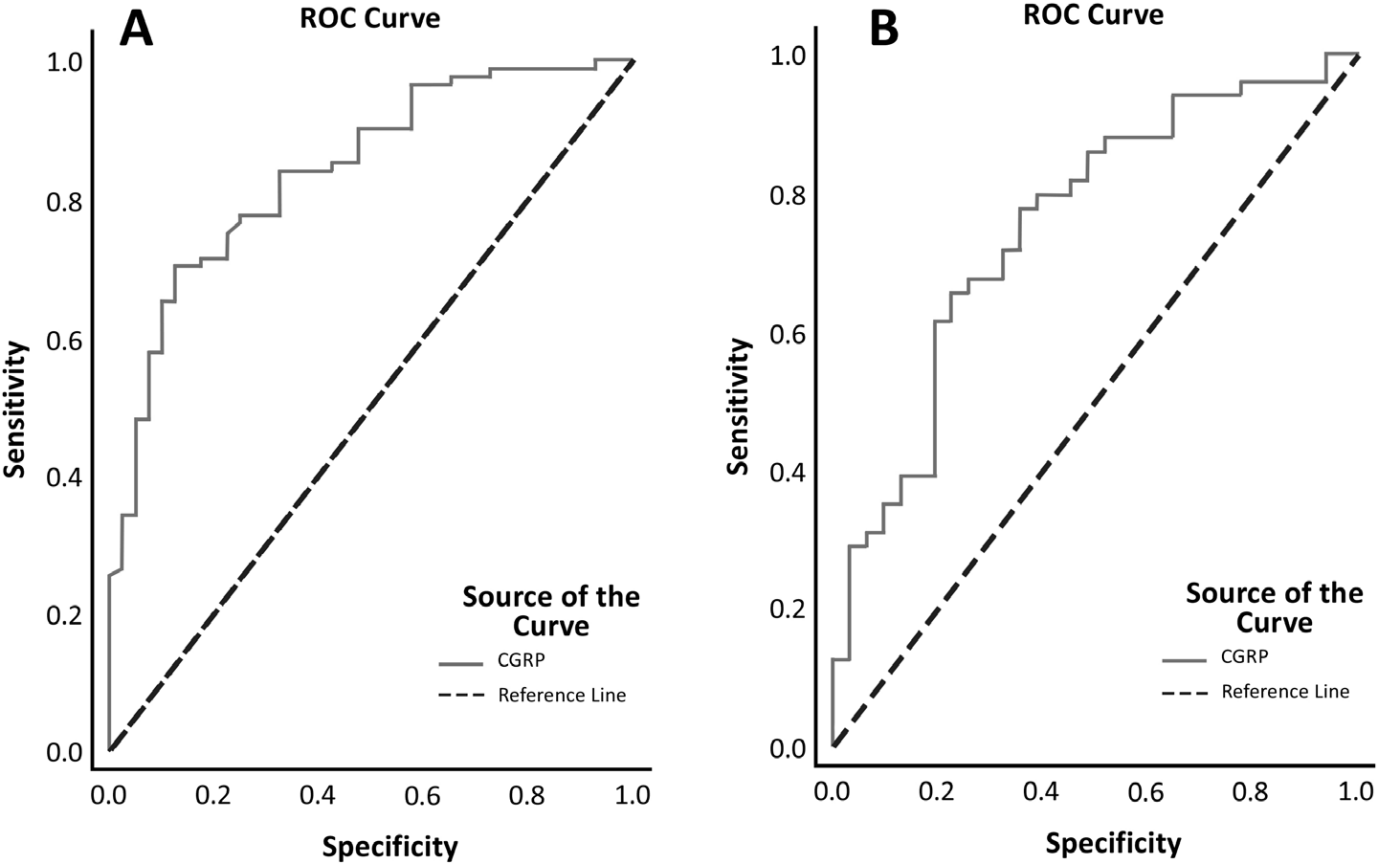
ROC Curve of Serum Calcitonin Gene-Related Peptide Levels (A), Serum CGRP Levels for Distinguishing DAS-28 Disease Activity Groups. (B).

The cut-off value for CGRP separating the patient and control groups was 61.78 pg/mL, with 70% sensitivity and 87.5% specificity (AUC: 0.839, *P* < .001) (Figure 1A). In the subgroup with CGRP levels exceeding 61.78 pg/mL, HAQ (*P* = .032), VAS-Patient (*P* = .005), VAS-Physician (*P* = .008), and RF positivity were notably elevated compared to those with CGRP levels at or below 61.78 pg/mL (Table 7). No statistically meaningful differences were observed between the groups regarding age, CRP, ESR, or positivity for anti-CCP and ANA.

The cut-off value for separating DAS-28 remission and active groups was 91.45 pg/mL, with 77.4% specificity and 65.3% sensitivity (AUC: 0.750, *P* < .001) (Figure 1B).

## Discussion

This cross-sectional study aimed to explore the association of serum CGRP concentrations with disease activity in patients diagnosed with RA. The findings revealed notably elevated serum CGRP levels in patients with RA compared to controls, underscoring a meaningful physiological distinction that merits further study. Furthermore, it was observed that the serum CGRP levels in seropositive patients with RA exhibited a statistically significant increase in comparison to their seronegative counterparts, indicating a potential biomarker distinction, while the CGRP levels in patients experiencing active levels of RA were markedly elevated in comparison to those observed in patients experiencing remission. This analysis underscores the importance of comprehending the role of CGRP in the pathophysiology of RA, as well as its potential implications for future therapeutic strategies and monitoring of disease progression. The cut-off value for the CGRP separating the patient and control groups was determined as 61.78 pg/mL, while the cut-off for the CGRP value separating remission and active patients with RA was determined as 91.45 pg/mL. The results indicated that CGRP, CRP, and ESR were determined to be associated with disease activity.

Despite effective treatment options, RA is still considered a disease that increases the rate of social disability by causing disability due to deformities and mortality due to extra-articular involvement.^19^ The facts that the etiopathogenesis of RA has still not been clearly understood, the disease affects a large number of patients, the biomarkers used in daily practice can be detected as negative in a significant portion of the patients, and there is no curative treatment although the RA treatment agents used today modify the disease, lead to the search for new biomarkers and alternative treatments.^20^

Calcitonin gene-related peptide is widely recognized as a highly effective vasodilator that exhibits significant pro-inflammatory properties, which are intricately involved in the complex mechanisms underlying the onset and progression of neuropathic pain as well as neurogenic inflammation,^21^ as highlighted in various scholarly articles. Calcitonin gene-related peptide plays a central part in complex pain pathways by modulating motor functions, sensory processing, and their integration within the central nervous system. As a result, CGRP antagonists have become key therapeutic agents for migraine management, mainly due to their effects on neurogenic inflammation and pain pathogenesis. Research has established that CGRP, along with its specific receptors, is not only present but also expressed in joint afferents, and notably, the levels of this peptide are significantly elevated in investigations concerning rheumatic diseases and various disorders of the musculoskeletal system.^22^ Specifically, a multitude of research investigations have established that serum concentrations of CGRP are significantly elevated in subjects experiencing muscular injuries,^23^ neck and shoulder pain,^24^ complex regional pain syndrome,^25^ neuropathic pain,^26^ as well as fibromyalgia,^19^ in contrast to those observed in healthy control subjects. Furthermore, the investigation conducted by Dong et al^10^ provided compelling evidence that serum levels of CGRP were similarly found to be increased in individuals suffering from knee osteoarthritis in comparison to healthy control cohorts.^13^ Furthermore, a relevant study on patients with RA reported that the mean serum CGRP concentration was significantly higher in this group compared to controls, measuring 0.82 ng/mL versus 0.31 ng/mL, respectively.^27^ In this study, serum concentrations of CGRP among individuals diagnosed with RA were observed to be elevated in comparison to healthy control subjects, similar to previous studies on inflammatory musculoskeletal diseases. Establishing a CGRP threshold capable of differentiating patients with RA from healthy controls could facilitate its use as a biomarker for RA. Analysis of the ROC curve determined a CGRP threshold of 61.78 pg/mL, yielding 70% sensitivity and 87.5% specificity. This cut-off value is considered a CGRP value that can statistically differentiate the disease in the diagnosis of RA.

The primary hypothesis of this study was that serum CGRP levels are elevated in patients with RA compared to controls and that these levels correlate meaningfully with disease activity in RA. To the authors’ knowledge, no previous studies have systematically investigated the relationship between serum CGRP levels and disease activity in patients with RA. Patients with RA were categorized according to disease activity and serostatus: seropositive (n = 40) and seronegative (n = 40), as well as active disease (n = 49) and remission (n = 31), based on DAS-28–ESR scores derived from the 28-joint count. Consistent with previous research, patients with active RA demonstrated markedly higher inflammatory markers such as ESR, CRP, RF, as well as increased VAS and HAQ scores assessed by both patients and physicians.^4,9,28-30^ The established significance of RF within the pathophysiological processes underlying RA, along with its recognized correlation with disease activity, is thoroughly substantiated.^31-33^ Significantly higher CGRP levels were detected in RF-positive patients compared to RF-negative ones, and a positive association between RF and CGRP was identified in this study. These findings imply that CGRP may have an important role in RA development and is closely connected to disease activity markers.

Elevated serum CGRP concentrations in the seropositive RA group relative to the seronegative group indicate a potential association between CGRP and the development of chronic synovitis and erosive arthritis. Consistent with earlier research, serum CGRP levels were found to be significantly elevated in patients with active RA compared to those in remission. High serum CGRP concentrations in subjects experiencing active RA are thought to play a role in neurogenic inflammation, thereby exacerbating pain and inflammatory responses. The logistic regression results demonstrated that increased CGRP, CRP, and ESR values correlate with an elevated risk of active disease. No established cut-off value currently exists for serum CGRP to assess disease activity in individuals diagnosed with RA. In the ROC analysis performed in this study, the cut-off value was obtained for CGRP as 91.4 pg/mL with 65.3% sensitivity and 77.4% specificity in determining disease activity. This cut-off value is considered to be a statistically acceptable CGRP value that can be used in the evaluation of disease activity in patients with RA.

Calcitonin gene-related peptide is thought to be a mediator of arthritis pain.^13^ It is well-established that the joints, along with osseous and muscular frameworks, are extensively supplied with sensory nerves that are immunoreactive to CGRP.^34^ In studies on osteoarthritis, which is characterized by joint pain, a relationship was reported between CGRP-immunoreactive innervation and joint pain.^35,36^ This study identified a significant association between patients’ serum CGRP levels and their DAS-28–ESR, HAQ, VAS-Patient, and VAS-Physician scores. Considering the CGRP-immunoreactive innervation of the synovium, higher serum CGRP levels could help identify patients experiencing pain due to RA. A study reported that intradiscal tumor necrosis factor alpha (TNF-α) inhibitor (etanercept) application reduced expression of CGRP in dorsal root ganglion neurons associated with injured intervertebral discs.^37^ Although CGRP levels were increased in RA patients, the lack of significant associations with CRP, ESR, and morning stiffness duration might be explained by the widespread use of bDMARD treatment among the study participants. Additionally, a positive relation was identified between CGRP value and DAS-28–ESR, HAQ, VAS-Patient, and VAS-Physician, whereas no correlation was obtained between CGRP and CRP, ESR, RA disease duration, and morning stiffness. The findings indicate that the influence of CGRP on the pain component in RA could be more pronounced.

Studies have shown that CGRP is affected by many factors, such as serotonin autoreceptors, glucocorticoids, retinoic acid, and vitamin D, which suppress CGRP gene expression.^38^ Calcitonin gene-related peptide levels and hormones vary between genders and different life stages, where the effect of the hormones plays a greater role for CGRP in women.^39^ A study of patients with knee osteoarthritis documented that CGRP expression was elevated in women compared to men, while it was positively correlated with the severity of pain in women.^40^ It could not be a coincidence that both CGRP levels and RA were observed to be higher in women, and CGRP may contribute to the development of RA. In this study, 75% of the participants in both the patient and control groups were women, so gender differences possibly affected the CGRP levels. Calcitonin gene-related peptide has both anti-inflammatory, pro-inflammatory, and anti-apoptotic effects.^41^ Baig and colleagues^42^ determined that methotrexate administration for adjuvant arthritis in rats decreased CGRP expression in the thymus. Most participants in this study were undergoing treatment for RA, which may have influenced both CGRP levels and disease activity in the RA group. Therefore, studies with larger sample sizes and comprehensive assessments of treatment regimens are needed to clarify the precise role of CGRP in RA patients.

This study holds particular significance by revealing, for the first time, a correlation between disease activity and serum CGRP levels in patients with RA, and by reporting elevated serum CGRP concentrations in these patients compared to healthy controls. This study’s limitations involve a limited sample size and the predominance of patients undergoing csDMARD and/or bDMARD therapy, underscoring the necessity for additional research to better understand how different treatments impact CGRP levels.

In conclusion, these findings indicate that increased serum CGRP levels may be involved in the pathophysiology of patients with RA, potentially correlating with disease progression, joint damage, and providing prognostic value in RA management. Nevertheless, to clarify CGRP’s precise role, further long-term studies with more subjects are essential.

## Figures and Tables

**Table 1.** Comparison of the Patient and Control Groups in Terms of Demographic Characteristics

		RA, n (%)	Control, n (%)	*P*
Gender	Female	60 (75)	30 (75)	.999^£^
	Male	20 (25)	10 (25)	
Marital status	Single	8 (10)	0 (0)	.051^¥^
	Married	72 (90)	40 (100)	
Profession	Those who do not work with physical strength	69 (86.3)	30 (75)	.126 ^£^
	Those who work with physical strength	11 (13.8)	10 (25)	
Educational status	Illiterate	5 (6.3)	0 (0)	**.029** ^□^
	Literate	7 (8.8)	0 (0)	
	Primary school	44 (55)	23 (57.5)	
	High school	14 (17.5)	5 (12.5)	
	University	10 (12.5)	12 (30)	
Smoke	Yes	20 (25)	13 (32.5)	.386^£^
	No	60 (75)	27 (67.5)	
RF	Positive	37 (46.3)		
	Negative	43 (53.8)		
Anti-CCP	Positive	36 (45)		
	Negative	44 (55)		
ANA	Positive	49 (61.3)		
	Negative	31 (38.8)		
Family history	Yes	36 (45)		
	No	44 (55)		
Disease onset	Slow	31 (38.8)		
	Middle	7 (8.8)		
	Acute	42 (52.5)		
Duration of morning stiffness	No	35 (43.8)		
	0-30 minutes	3 (3.8)		
	30-59 minutes	15 (18.8)		
	>1 hour	27 (33.8)		
DAS-28	Remission	31 (38.8)		
	Low disease activity	5 (6.3)		
	Medium disease activity	18 (22.5)		
	High disease activity	26 (32.5)		
HAQ	Mild difficulties and moderate disability	49 (61.3)		
	Moderate to severe disability	25 (31.3)		
	Severe difficulties and very severe disability	6 (7.5)		
Treatment	No	6 (7.5)		
	csDMARD	28 (35)		
	bDMARD	19 (23.8)		
	csDMARD + bDMARD	27 (33.8)		
**Mean ± SD, Median (Interquartile Range)**				
Age* (years)		51.2 ± 9.9, 53 (15.5)	50.9 ± 7.1, 52 (11)	.464^&^
DAS-28*		3.8 ± 1.9, 4.1 (3.3)		
HAQ*		0.6 ± 0.7, 0.4 (1.3)		
VAS-Patients^#^		3 (5)		
VAS-Physician^#^		3 (5)		
Number of sensitive joints*		7.8 ± 9.4, 2.5 (14)		
Number of swollen joints*		2.0 ± 4.7, 4.1 (3.3)		

ANA, antinuclear antibody; Anti-CCP, anti-cyclic citrullinated peptide; bDMARD, biological DMARD; CGRP, calcitonin gene-related peptide; CRP, C-reactive protein; csDMARD, conventional synthetic DMARD; DAS-28, Disease Activity Score 28; DMARDs, disease-modifying antirheumatic drugs; ESR, erythrocyte sedimentation rate; HAQ, Health Assessment Questionnaire; RA, rheumatoid arthritis; RF, rheumatoid factor; VAS, Visual Analog Scale.

*Mean ± SD, Median (interquartile range).

^#^Median (interquartile range).

^&^Mann–Whitney *U*-test.

^£^Pearson’s chi-square test.

^□^Fisher–Freeman–Halton test.

^¥^Fisher’s exact test.

**Table 2.** Comparison of Calcitonin Gene-Related Peptide Levels of Patient and Control Groups

	RA*	Control*	*P* ^&^
CGRP, pg/mL	129.5 ± 103.2, 91.1 (112.5)	48.4 ± 28.0, 40.8 (25.7)	**<.001**
BMI	30.0 ± 6.1, 28.8 (6.6)	25.5 ± 5.2 25.1, (8.6)	**<.001**
CRP	1.1 ± 1.7 0.5 (1.1)	0.2 ± 0.1 0.2 (0.2)	**<.001**
ESR	25.5 ± 19.4 22 (26.5)	13.5 ± 7.7, 11 (10.5)	**.002**
CGRP Levels*, pg/mL			
Seropositive	154 ± 116, 118.7 (145.7)		**.017**
Seronegative	105.1 ± 83.2, 66.1 (69.7)		
**RF**			
Positive	156.2 ± 116.5, 122.1 (132.2)		**.012**
Negative	106.5 ± 85.1, 64 (72.2)		
**Anti-CCP**			
Positive	149.3 ± 120.1, 113.7 (125.8)		.128
Negative	113.4 ± 85.1, 79.5 (99.9)			
**ANA**			
Positive	126.5 ± 113, 83.9 (111.8)		.311
Negative	134.4 ± 87.1, 111.5 (175.1)		
**Smoke**			
Yes	129.5 ± 127.8, 83.9 (125.1)		.365
No	129.5 ± 94.9, 96.7 (107.7)		
**Treatment**			
No	166.1 ± 156, 105.1 (96.9)		.590
csDMARD	155.5 ± 126.1, 108.3 (183.2)		
bDMARD	115.4 ± 82.4, 90.2 (123.1)		
csDMARD + bDMARD	104.4 ± 68.3, 75.4 (83.3)		

ANA, antinuclear antibody; Anti-CCP, anti-cyclic citrullinated peptide; bDMARD, biological DMARD; CGRP, calcitonin gene-related peptide; CRP, C-reactive protein; csDMARD, conventional synthetic DMARD; DAS-28, Disease Activity Score 28; DMARDs, disease-modifying antirheumatic drugs; ESR, erythrocyte sedimentation rate; HAQ, Health Assessment Questionnaire; RA, rheumatoid arthritis; RF, rheumatoid factor.

*Mean ± SD, Median (interquartile range).

^&^Mann–Whitney *U*-test.

**Table 3.** Comparison of Rheumatoid Arthritis Patients' Findings According to Disease Activity Score 28 Groups

	DAS-28		*P*
	Moderate Disease Activity	High Disease Activity	
Age* (years)	51.4 ± 8.3, 51 (11)	52.2 ± 8.8, 53.5 (15)	.752^€^
CGRP*	184.5 ± 124.7, 175.3 (198.1)	152.7 ± 112.7, 118.7 (105)	.543^&^
CRP *	1.6 ± 2.3, 0.6 (1.7)	1.6 ± 1.8, 1.1 (1.9)	.424^&^
ESR *	29.4 ± 20.9, 27 (32)	34.3 ± 20.7, 31 (22)	.459^&^
HAQ *	0.7 ± 0.5, 0.6 (0.9)	1.3 ± 0.5, 1.4 (0.6)	**<.001** ^€^
VAS-Patients^#^	3.5 (1)	6.5 (2)	**<.001** ^&^
VAS-Physician^#^	3.5 (1)	6 (2)	**<.001** ^&^
**RF**	**n (%)**	**n (%)**	
Positive	8 (44.4)	16 (61.5)	.263^£^
Negative	10 (55.6)	10 (38.5)	
**Anti-CCP**			
Positive	7 (38.9)	15 (57.7)	.220^£^
Negative	11 (61.1)	11 (42.3)	
**ANA**			
Positive	7 (38.9)	19 (73.1)	**.023** ^£^
Negative	11 (61.1)	7 (26.9)	

ANA, antinuclear antibody; Anti-CCP, anti-cyclic citrullinated peptide; CGRP, calcitonin gene-related peptide; CRP, C-reactive protein; DAS-28, Disease Activity Score 28; DMARDs, disease-modifying antirheumatic drugs; ESR, erythrocyte sedimentation rate; HAQ, Health Assessment Questionnaire; RA, rheumatoid arthritis; RF, rheumatoid factor.

*Mean ± SD, Median (interquartile range).

^#^Median (interquartile range).

^&^Mann–Whitney *U*-test.

^£^Pearson’s chi-square test.

^€^Independent samples *t*-test.

**Table 4.** Comparison of Findings of Remission and Active Rheumatoid Arthritis Patients According to Disease Activity Score-28 Groups

	**DAS-28**		***P***
	**Remission**	**Active**	
CGRP*	82.5 ± 60.3, 59.4 (48.7)	159.3 ± 113.7, 117.8 (158)	**<.001^&^**
CRP*	0.5 ± 0.9, 0.3 (0.6)	1.5 ± 1.9, 0.9 (1.8)	**.001^&^**
ESR*	14.1 ± 10.5, 10 (17)	32.7 ± 20.3, 30 (28)	**<.001^&^**
HAQ*	0.1 ± 0.4, 0 (0)	1 ± 0.6, 1 (1.1)	**<.001^&^**
VAS-Patients^#^	0 (1)	4 (4)	**<.001^&^**
VAS-Physician^#^	0 (1)	4 (3)	**<.001^&^**
**RF**	**n (%)**	**n (%)**	
Positive	9 (29)	28 (57.1)	**.014^£^**
Negative	22 (71)	21 (42.9)	
**Anti-CCP**			
Positive	11 (35.5)	25 (51)	.174^£^
Negative	20 (64.5)	24 (49)	
**ANA**			
Positive	21 (67.7)	28 (57.1)	.343^£^
Negative	10 (32.3)	21(42.9)	

ANA, antinuclear antibody; Anti-CCP, anti-cyclic citrullinated peptide; CGRP, calcitonin gene-related peptide; CRP, C-reactive protein; DAS-28, Disease Activity Score 28; DMARDs, disease-modifying antirheumatic drugs; ESR, erythrocyte sedimentation rate; HAQ, Health Assessment Questionnaire; RA, rheumatoid arthritis; RF, rheumatoid factor.

*Mean ± SD, Median (interquartile range).

^#^Median (interquartile range).

^&^Mann–Whitney *U*-test.

^£^Pearson’s chi-square test.

^€^Independent samples *t*-test.

**Table 5.** Factors Affecting Disease Activity Score-28–Erythrocyte Sedimentation Rate Activity

	B	SE	Wald	*df*	*P*	OR	95% CI
CGRP and CRP							
CGRP	0.013	0.004	9.713	1	**.002**	1.013	1.005-1.022
CRP	0.975	0.384	6.460	1	**.011**	2.651	1.250-5.623
Constant	−1.8410	0.593	9.311	1	**.002**	0.164	
CGRP and ESR							
CGRP	0.014	0.004	10.090	1	**.001**	1.014	1.005-1.023
ESR	0.092	0.025	13.821	1	**<.001**	1.097	1.045-1.152
Constant	−3.102	0.806	14.792	1	**<.001**	0.045	

B, regression coefficient; CGRP, calcitonin gene-related peptide; CRP, C-reactive protein; ESR, erythrocyte sedimentation rate; OR, odds ratio; SE, standard error.

**Table 6.** Correlation Between Patients’ Calcitonin Gene-Related Peptide Values and Other Values

		CGRP
DAS-28	***r***	0.376
	***P***	**.001**
HAQ	***r***	0.303
	***P***	**.006**
CRP	***r***	0.013
	***P***	.911
ESR	***r***	0.128
	***P***	.259
RA time-onset	***r***	0.025
	***P***	.826
VAS-Patients	***r***	0.387
	***P***	**<.001**
VAS-Physician	***r***	0.376
	***P***	**.001**
Age (years)	***r***	0.110
	*** P***	.332
Duration of morning stiffness	***r***	0.220
	*** P***	.050

CGRP, calcitonin gene-related peptide; CRP, C-reactive protein; DAS-28, Disease Activity Score 28; ESR, erythrocyte sedimentation rate; HAQ, Health Assessment Questionnaire; RA, rheumatoid arthritis; VAS, Visual Analog Scale; *r,* Spearman correlation coefficient.

**Table 7.** Comparison of Some Parameters in Rheumatoid Arthritis Patients According to the Cutoff Values of Calcitonin Gene-Related Peptide

	CGRP Cutoff Groups		*P*
	≤61.78	>61.78	
Age* (years)	50.2 ± 7.2, 48.5 (11.5)	51.7 ± 10.8, 54 (18)	.246
CRP*	1.1 ± 1.3, 0.5 (1.2)	1.2 ± 1.8, 0.6 (1.1)	.648
ESR*	22 ± 17, 16.5 (22)	27 ± 203, 24 (28.5)	.413
HAQ*	0.4 ± 0.6, 0 (0.5)	0.8 ± 0.7, 0.6 (1.4)	**.032**
VAS-Patients^#^	1 (3)	4 (4.5)	**.005**
VAS-Physician^#^	0.5 (3)	4 (5.5)	**.008**
**RF**	**n (%)**	**n (%)**	
Positive	5 (20.8)	32 (57.1)	**.003**
Negative	19 (79.2)	24 (42.9)	
**Anti-CCP**			
Positive	7 (29.2)	29 (51.8)	.062
Negative	17 (70.8)	27 (48.2)	
**ANA**			
Positive	15 (62.5)	34 (60.7)	.881
Negative	9 (37.5)	22 (39.3)	

ANA, antinuclear antibody; Anti-CCP, anti-cyclic citrullinated peptide; CGRP, calcitonin gene-related peptide; CRP, C-reactive protein; DAS-28, Disease Activity Score 28; DMARDs, disease-modifying antirheumatic drugs; ESR, erythrocyte sedimentation rate; HAQ, Health Assessment Questionnaire; RA, rheumatoid arthritis; RF, rheumatoid factor.

*Mean ± SD, Median (interquartile range).

^#^Median (interquartile range).

## Data Availability

The data that support the findings of this study are available on request from the corresponding author.
